# Exercise Preconditioning Plays a Protective Role in Exhaustive Rats by Activating the PI3K-Akt Signaling Pathway

**DOI:** 10.1155/2020/3598932

**Published:** 2020-01-21

**Authors:** Jingjing Li, Peng Xu, Yang Wang, Zheng Ping, Xuebin Cao, Yu Zheng

**Affiliations:** ^1^Department of Cardiology, The Hospital of the 82nd Group Army, Baoding 071000, Hebei, China; ^2^Department of Clinical Pharmacy, The Hospital of the Group 82nd Army, Baoding 071000, Hebei, China; ^3^School of Life Sciences, Tianjin Polytechnic University, Tianjin 300387, China

## Abstract

**Objective:**

To investigate whether exercise preconditioning (EP) protects the rat heart from exhaustive exercise- (EE-) induced injury by inducing the PI3K-Akt signaling pathway.

**Methods:**

84 male Sprague-Dawley rats were randomly divided into 6 groups (*n* = 14 rats per group): control group (Con), exhaustive exercise group (EE), exercise preconditioning group (EP), exercise preconditioning + exhaustive exercise group (EP + EE), LY294002 (PI3K inhibitor) + exercise preconditioning + exhaustive exercise group (LY + EP + EE), and LY294002 group (LY). The Con and LY did not exercise. The remaining groups were subjected to treadmill running. The structure of myocardial tissue and serum biomarkers of myocardial injury were observed. Hemodynamic parameters were recorded with a pressure-volume catheter. TUNEL assay was used to detect the apoptosis of cardiac myocytes, and the level of mitochondrial membrane permeability transforming pore (mPTP) in myocardium was evaluated using ELISA. Pathway and apoptosis-related proteins in myocardium were assessed using western blotting.

**Results:**

Compared to the Con group, the EE group showed remarkable myocardial injury, such as cardiac dysfunction and myocardial apoptosis. Compared to the EE group, the injuries in the EP + EE group were improved. EP increased the PI3K-Akt signaling pathway and regulated Bcl-2 family to decrease the mPTP openness level. However, the cardioprotective effects of EP were attenuated when pretreated with the LY294002.

**Conclusions:**

EP protected the heart from EE-induced injury, and it may improve the cardiac function and reduce the cardiomyocyte apoptosis by activating the PI3K-Akt signaling pathway.

## 1. Introduction

Acute overload exercise, such as exhaustive exercise (exercise intensity or duration exceeding the body's limit, EE), has adverse effects on the heart. It causes EE-induced myocardial injury, which is manifested as pathological changes of myocardium, abnormal markers of myocardial injury, cardiac function decreases, and even sudden death of sports occurs. Exercise-induced heart injury is more common in people who are often engaged in high-intensity work, such as athletes, soldiers, and other groups. However, exercise preconditioning (EP) is a pragmatic countermeasure to protect against cardiac injury [1]. The studies have stated the powerful beneficial effects of EP [2–4]. Therefore, we want to study the effects and related mechanisms of EP on improving cardiac injury caused by EE.

Several factors have been proposed as mediators of EP's protection, including an increase in miR-17-3p, Ca^2+^-handling mechanisms, and heat shock protein upregulation in the myocardium [5–8]. However, the mechanisms responsible for exercise-induced cardioprotection are not well established at present. The phosphatidylinositol 3 kinase-protein kinase B (PI3K-Akt) signaling pathway plays an important role in the exercise-induced cardiac protection mechanism [9–11]. Huang et al. found that EP activated the PI3K-Akt signaling pathway to maintain cardiac morphology and function. And, p-Akt appeared to promote the anti-apoptosis pathway. Akt is one of the major upstream signal proteins in the Bcl-2 family [12]. Bcl-2 family directly controls the mitochondrial pathway of apoptosis [13]. Bcl-2 protein family influences the apoptosis of cardiomyocytes by regulating the state of mitochondrial membrane permeability transforming pore (mPTP) [1]. It has been found that the PI3K-Akt signaling pathway affected the apoptosis of cardiomyocytes by regulating the open state of mPTP [14, 15]. Enhanced open level of mPTP contributes to the increase of mitochondrial membrane permeability; therefore, cytochrome C (cyto-c) and other apoptotic factors are released into the cytoplasm to form an apoptotic complex, and it is responsible for activating caspase-9, which further activates caspase-3 and executes the apoptotic program [16]. Our previous experiments confirmed that exhausted swimming exercise increased the apoptosis level through increasing the Bax/Bcl-2 ratio and increased the expression of cyto-c, caspase-3, and caspase-9 which was related to the mitochondrial apoptosis pathway [17]. However, the effect of EE on the PI3K-Akt signaling pathway and the association of the PI3K-Akt signaling pathway activated by EP and cardiac protection and resistance to the injury of EE remain unclear. To gain insight into the potential mechanisms responsible for exercise-induced cardioprotection against EE-induced apoptosis, we further evaluated the role of p-PI3K and p-Akt in this beneficial effect of exercise. We hypothesize that EP can protect heart to relieve the cardiac injury induced by EE, and the protection is possibly triggered by the PI3K-Akt signaling pathway.

This study can be used as a reference for the scientific formulation of exercise preconditioning training programs and for improving the prevention and treatment of exercise-induced heart injury.

## 2. Materials and Methods

### 2.1. Establishment and Grouping of Animal Models

84 male Sprague-Dawley rats (200–220 g) were provided by Sibeifu Biotechnology Company Limited (Beijing, China). Animals were housed with the indoor temperature maintained at 20 to 22°C, received a 12/12 h light-dark cycle, and fed with standard rodent mash and water ad libitum. All experiments were conducted in compliance with the guide for the Care and Use of Laboratory Animals and reviewed and approved by the Ethics Committee for the Use of Experimental Animals at No. 252 Hospital of the Chinese People's Liberation Army.

All animals were subjected to a week of treadmill running (10–15 m/min for 15 min, in 0° slope). After the adaptive treadmill training, they were randomly divided into 6 groups (*n* = 14 rats per group): control group (Con group), exhaustive exercise group (EE group), exercise preconditioning group (EP group), exercise preconditioning + exhaustive exercise group (EP + EE group), LY294002 (PI3K inhibitor) + exercise preconditioning + exhaustive exercise group (LY + EP + EE), and LY294002 group (LY group). Owing to the Millar catheter procedure was an invasive test, it may affect other experimental results, so each group of the rats were divided into two parts: one part was used for the pressure volume catheter detection of cardiac function (*n* = 8 animals per group) and the remaining animals (*n* = 6 animals per group) were used to collect Serum and myocardial specimens. The exercise training protocol referenced to Domenech et al. [18] and Lennon's et al. [19]. The Con group did not run on the treadmill. The EE group was subjected to consecutive running in 0° slope and 25–30 m/min until exhausted. The training speed was based on the acceptable maximum intensity of individual. The criteria of exhaustion was the running posture of the rats changing from the original push-off to the push-down and remaining at the back of the track; even under the influence of sound and light stimulation, the rats still could not continue running. The EP group was in 0° slope and 30 m/min for three periods of 15 min running and 5 min of intermittent recovery; this process needs 60 min per day and the exercise training proceeds for six days per week, a total of 3 weeks. EP + EE group included an EE period after the EP period. LY + EP + EE group rats were given intraperitoneal injection of LY294002 (Selleck, USA, 10 mg/kg, dissolved in 100% DMSO) 30 min before exercise training, and the training method was same as that of the EP + EE group [20]. The LY group was given intraperitoneal injection of LY294002 (10 mg/kg). All exercise groups underwent prior warm up in 0° slope and at an initial velocity of 10 m/min for 5 min, and the velocity gradually increased up to the target. EP group animals were tested immediately after EP training. The exhausted animals were tested immediately after the exhaustion was reached.

### 2.2. Structure of Myocardium Was Observed by Optical Microscopy and Transmission Electron Microscopy (TEM)

All rats were anesthetized with pentobarbital sodium (40 mg/kg, intraperitoneal injection). The hearts were quickly removed and washed with cold saline. Myocardial tissue of the left ventricle was collected for light microscopy analysis. The tissue was fixed with 10% formaldehyde, paraffin-embedded, sectioned, dehydrated with different concentration gradients of alcohol, stained with HE, and observed by the light microscopy (Olympus, Japan).

Myocardial tissue of the left ventricle was harvested for TEM analysis. Briefly, tissues (1 mm^3^) were fixed with fresh prepared 4% glutaraldehyde for 5 h, washed using phosphate buffer, then postosmicated in 1% osmium tetroxide for 2 h, dehydrated with gradient acetone, embedded, sectioned, electron stained with uranyl acetate and lead citrate, and then observed by transmission electron microscopy (HT7700, Hitachi, Japan).

### 2.3. Enzyme-Linked Immunoassay for CK-MB and cTn-I Levels in the Serum of Exhausted Rats

The blood sample was collected from the inferior vena cava, then centrifuged at 2000 r/min for 25 min, and the supernatant was collected and stored at −80°C. The contents of cardiac troponin I (cTn-I) and CK-MB in serum were detected by Rat cTn-I ELISA kit (Kmaels, China) and Rat CK-MB ELISA kit (Kmaels, China), which strictly followed the manufacturer's instructions. The OD values in each hole at 450 nm were measured by microplate micrograph (Thermo, USA), and the standard curve was drawn to calculate the sample concentration.

### 2.4. Determination of Cardiac Function Parameters with a Pressure Volume Catheter

The rats were weighted and anesthetized with pentobarbital sodium (40 mg/kg, intraperitoneal injection) and fixed on the operating table in supine position. After endotracheal intubation, we separated the right carotid artery and calibrated the pressure with MPVS control software. The Millar catheter (SPR-838, Millar, USA) was inserted into the left ventricle from the right carotid artery. The left ventricular pressure volume waveforms of the anesthetized rats were recorded with Chart7 software in real-time. The basic waveform was recorded for 10 min. A ventral midline incision was performed on the abdomen, the inferior vena cava was occluded, and the waveform was recorded. 30% NaCl solution (30 *μ*l) was injected in the left jugular vein, and the pressure-volume waveform was recorded. Then, the catheter tips were submerged in the holes of a calibration cuvette which was filled with fresh heparinized warm blood, respectively, and the conductance changes in the volume channel were recorded, and finally the volume can be calculated. The parameters, stroke work (SW), cardiac output (CO), stroke volume (SV), heart rate (HR), end-systolic volume (Ves), end-diastolic volume (Ved), end-systolic pressure (Pes), end diastolic pressure (Ped), ejection fraction (EF), peak rate of pressure rise (dp/dt max), peak rate of pressure decline (dp/dt min), potential energy (PE), cardiac efficiency (CE), end-systolic pressure volume relationship (ESPVR), end-diastolic pressure volume relationship (EDPVR), and relaxation time constant (Tau), were detected.

### 2.5. Terminal Deoxynucleotidyl Transferase-Mediated dUTP-Biotin Nick End Labeling Assay (TUNEL) Technique Was Used to Detect Apoptosis Index of Cardiomyocytes

Apoptosis in cardiomyocytes was determined with TUNEL technique. TUNEL staining was performed using the InSitu Cell Death Detection kit (Roche, SUI) according to the manufacturer's instructions. Five visual fields of heart sections were randomly selected in each animal, and TUNEL-positive cells were counted. Apoptosis indices are expressed as the percentage of positive cells in the total number of cardiomyocytes in the field.

### 2.6. Enzyme-Linked Immunoassays for the Open Level of mPTP in the Myocardium of Exhausted Rats

The open level of myocardial mPTP was measured by Rat mPTP ELISA Kit (Sbjbio, China), and the operation process was in accordance with the manufacturer's instructions of the kits.

### 2.7. Western Blotting Analyses of p-PI3K, p-Akt, Bad, Bcl-2, Bax, and Caspase-3 Levels in the Left Ventricular Myocardium

The left ventricle wall was collected and rapidly frozen in liquid nitrogen and stored at −80°C. The heart was removed from a −80°C freezer, and the left ventricular myocardial tissue was sheared on ice. The myocardial tissue was homogenized to directly collect the supernatant liquor. The protein sample was mixed with buffer and heated to denaturation, and then SDS-PAGE was added. The sample was transferred to a polyvinylidene fluoride (PVDF) membrane, sealed with a sealing solution for 1 h, and then incubated overnight at 4°C with the corresponding primary antibodies, including p-PI3K antibody (Bioss, China, 1 : 600), p-Akt antibody (Bioworld, China, 1 : 600), Bad antibody (Bioss, China, 1 : 600), Bcl-2 antibody (Abcam, UK, 1 : 500), Bax antibody (Abcam, UK, 1 : 1000), Caspase-3 antibody (Santacruz, USA, 1 : 400), and *β*-actin antibody (ZSGB-BIO, China, 1 : 1000). The reaction membrane was incubated with secondary goat anti-rabbit IgG antibody (ZSGB-BIO, China, 1 : 3000) at room temperature for 1 h. The ECL luminescence kit (Santacruz, USA) was used for colorimetric detection. Images were scanned and measured in terms of the grayscale values by the gel imaging system (UVP, USA).

### 2.8. Statistical Analysis

SPSS 20.0 statistical software was used to analyze the measurement data, and the results were expressed as means ± SD. A homogeneity test was performed, and one-way analysis of variance (ANOVA) was used for the comparison multiple groups. Comparisons of mean values between two groups were performed using the LSD test if the variance was equal or Dunnett's T3 method if the variance was unequal. A correlation analysis was performed by calculating Pearson's correlation coefficients. *P* < 0.05 was considered statistically significant.

## 3. Results

### 3.1. EP Provides a Protective Effect on Myocardial Structure of Exhausted Rats

To evaluate the extent of myocardial structure injury caused by exercise, we used light microscopy and TEM to observe the structure changes.

**Figure 1:**
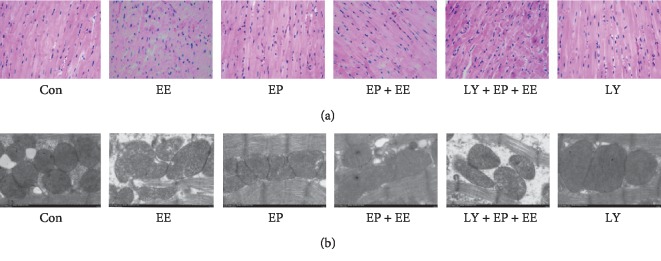
EP provides protective effects on myocardial structure of exhausted rats. Con: sedentary control group; EE: exhaustive exercise group; EP: exercise preconditioning group; EP + EE: exercise preconditioning + exhaustive exercise group; LY + EP + EE : LY294002 (PI3K inhibitor) + exercise preconditioning + exhaustive exercise group; LY: LY294002 group. (a) Light microscopy showed changes in myocardial microstructure among the six groups. Original magnification was ×400. Images showed disorganization and breakage of myocardial fiber, cardiomyocytes degeneration and necrosis, and interstitial substance with edema in the EE group. The LY + EP + EE group and the EP + EE group showed EE-like microstructure damages, but the EP + EE group was slighter. The EP group showed that myocardial fibers were organized, thickened, and slightly swollen. Con and LY groups showed normal myocardial microstructure. (b) Transmission electron microscopy showed changes in myocardial ultrastructure. Magnification was ×15 K; bar = 1.0 *μ*m. Con and LY groups showed normal myocardial ultrastructure. The EP group showed that mitochondria were slightly swollen morphologically. The EE group myocardial fibers fractured, the mitochondria were swollen morphologically, the mitochondrial membrane structure partially disappeared, and the mitochondrial cristae fused and even disappeared. In the EP + EE group, myocardial fiber rupture was less, the mitochondria were swollen, and partial mitochondrial membrane and mitochondrial cristae become blurred. EE-like ultrastructural damages were observed in the LY + EP + EE group.

In the Con group, myocardial fibers were arranged neatly and the cardiomyocyte membranes showed integrity. The EP group showed myocardial fiber thickening and slight swelling. The myocardial fibers of EE group exhibited disorganization, breakage, degeneration, and necrosis and had interstitial edema; the cardiomyocyte membranes were also damaged. However, the myocardial fibers of the EP + EE group did not show severe damage. Under the influence of LY294002 (PI3K inhibitor), EE-like microstructure damages were observed in the LY + EP + EE group. We could see the EE-induced microstructure alterations were significantly suppressed by EP, but the protective effect of EP was partly attenuated by LY294002 (Figure 1(a)).

The myocardial ultrastructure of the Con group apparently displayed normal morphology and was mainly characterized by well-ranged myocardial fibers and normal shape, size, and amount of mitochondria. The morphology of the mitochondria in the EP group was larger and elliptic or spindle in shape. However, we could see myocardial fibers of the EE group fractured and the mitochondria showed severe damage. In the EE group, the mitochondria were swelling, the mitochondrial membrane partially disappeared, and the mitochondrial cristae fused and even disappeared. These injurious alterations were significantly relieved in the EP + EE group, myocardial fiber rupture was less, the morphology of the mitochondria was slightly swelling, and mitochondrial membrane and mitochondrial cristae were just partial and became blurred. Under the influence of LY294002, EE-like ultrastructural damages were observed in the LY + EP + EE group (Figure 1(b)).

### 3.2. EP Can Reduce the Levels of Serum CK-MB and cTn-I Induced by EE

**Figure 2:**
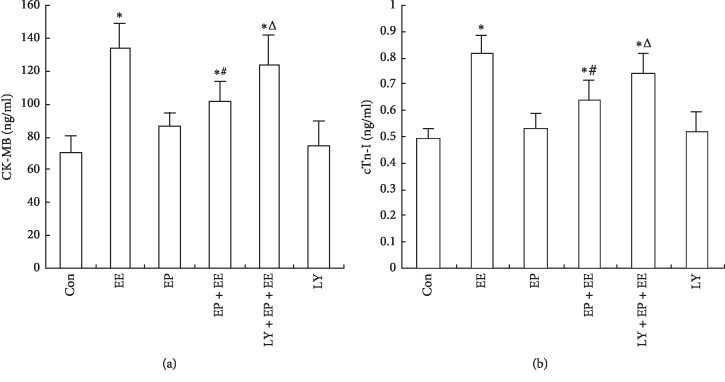
EP reduced the level of CK-MB and cardiac troponin I (cTn-I) in serum of exhausted rats (*x* ± *s*, *n* = 6). (a) The serum level of CK-MB in each group determined by ELISA. (b) The serum level of cTn-I in each group indicated by ELISA. For groups, see the footnote in Figure 1. ^*∗*^*P* < 0.05 compared with the Con group. ^#^*P* < 0.05, EP + EE group compared with the EE group. ^Δ^*P* < 0.05, LY + EP + EE group compared with the EP + EE group.

As an indicator, the level of CK-MB and cTn-I in serum can reflect myocardial injury. Compared with the Con group, the levels of CK-MB and cTn-I in the EE group, EP + EE group, and LY + EP + EE group were significantly increased (*P* < 0.05). Compared to the EE group, the EP + EE group exhibited a significant decrease and no differences in the LY + EP + EE group. The levels of CK-MB and cTn-I in LY + EP + EE group were significantly higher than those in the EP + EE group (*P* < 0.05). The levels of LY group and EP group were not statistically different from those of the Con group (*P* > 0.05) (Figure 2).

### 3.3. Effect of EP on the Cardiac Function Parameters of Exhausted Rats

**Figure 3:**
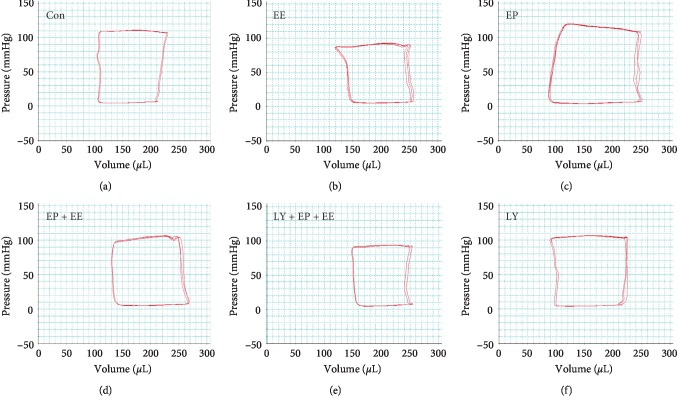
Original recording showing the pressure-volume loops. 0the footnote in Figure 1. Compared with the Con group, the EE group, EP + EE group, and LY + EP + EE group showed reduced SV along with increased Ves and increased Ved; thus, the position of P-V loops shifted to the right and the EP group showed decreased Ves and decreased Ved; thus, the position of P-V loops shifted to the left.

The cardiac function was measured by hemodynamic parameters. The EE group showed the decreased width in P-V loops, and the original diagram reflects reduced SV along with increased Ves and increased Ved. EF, CO, d*p*/d*t*_max_, and ESPVR all decreased significantly; this suggested deteriorated systolic performance in rats after EE. Significant decrease in −d*p*/d*t*_min_ and increase in EDPVR and Tau indicated impaired diastolic function. The results of SW and CE showed significant decrease, and PE was significantly increased, which meant deteriorated cardiac mechanoenergetics. However, these damaging changes could be mitigated by EP as we observed in the EP + EE group. In this group, CO, SV, Pes, EF, d*p*/d*t*_max_, ESPVR, −d*p*/d*t*_min_, SW, and CE all increased significantly, and Ves, Ved, EDPVR, Tau, and PE all decreased obviously. The changes of cardiac function parameters indicated that EP provided cardioprotective function to EE. In the LY + EP + EE group, the data showed that CO, SV, EF, d*p*/d*t*_max_, ESPVR, −d*p*/d*t*_min_, SW, and CE decreased significantly; Ves and Tau were significantly increased; these suggested that the cardiac function aggravated again. As a PI3K inhibitor, LY294002 suppressed the cardiac function protection of EP to EE (Figure 3; Table 1).

### 3.4. EP Reduced the Cardiomyocyte Apoptosis in the Myocardium of Exhausted Rats

**Figure 4:**
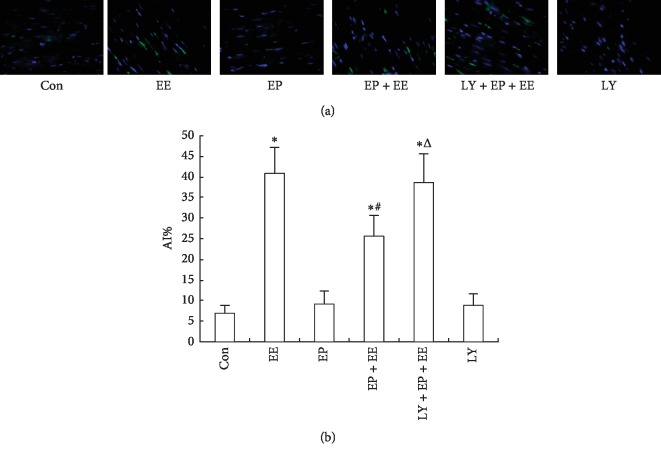
EP reduced the cardiomyocyte apoptosis in the myocardium of exhausted rats (*x* ± *s*, *n* = 6). The apoptosis of cardiomyocyte was detected by TUNEL. Original magnification was ×400. For groups, see the footnote in Figure 1. (a) TUNEL staining showed the normal cells in blue and the positive cells in green. Few green stains in the Con, EP, and LY groups were observed. In the EE, EP + EE, and LY + EP + EE groups, a significant portion of the tissues were stained green. (b) EP reduced the apoptosis index (AI) in the myocardium of exhausted rats. AI was the percentage of positive cells in the total number of cells. ^*∗*^*P* < 0.05 compared with the Con group. ^#^*P* < 0.05, EP + EE group compared with the EE group. ^Δ^*P* < 0.05, LY + EP + EE group compared with the EP + EE group.

TUNEL staining showed the normal cells were blue, whereas the positive cells were green and the percentage of positive cells in the total number of cells indicated the apoptosis index (AI). Few green stains were observed in the Con, EP, and LY groups. However, significant portions of the tissues in the EE, EP + EE, and LY + EP + EE groups were stained green after EE, which indicated increased apoptosis. TUNEL analysis showed significant increase in the EE, EP + EE, and LY + EP + EE groups with AI. But the AI of the EP + EE group was obviously lower than that of the EE group, which might be due to the protective effect of EP. The AI of LY + EP + EE group was significantly higher than the EP + EE group, which meant that the protective effect of EP could be weakened by LY294002 (Figure 4).

### 3.5. Effect of EP on the Open Level of mPTP in the Myocardium of Exhausted Rats

**Figure 5:**
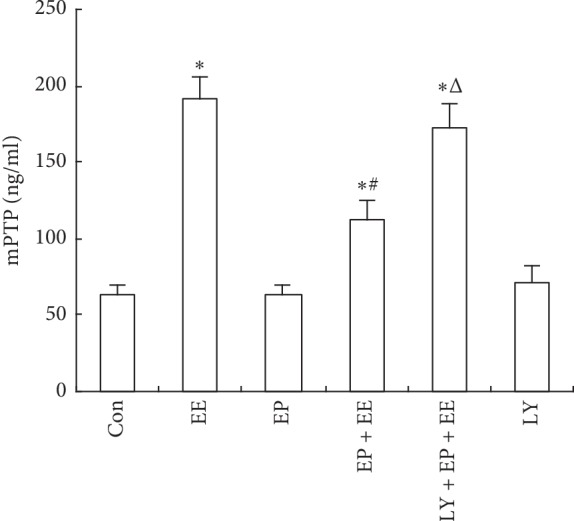
EP reduced the open level of myocardial mitochondrial permeability transition pore (mPTP) in the myocardium of exhausted rats (*x* ± *s*, *n* = 6). For groups, see the footnote in Figure 1. ^*∗*^*P* < 0.05 compared with the Con group. ^#^*P* < 0.05, EP + EE group compared with the EE group. ^Δ^*P* < 0.05, LY + EP + EE group compared with the EP + EE group.

The open level of mPTP was used to evaluate the station of mitochondrial membrane permeability. EE, EP + EE, and LY + EP + EE groups all showed a significant increase in the open level of mPTP compared to the Con group. The EP + EE group showed a significant decrease in the open level of mPTP compared to the EE group. The mPTP open level in the LY + EP + EE group was significantly higher than that of EP + EE group. It showed no significant difference in mPTP open levels among Con, EP, and LY groups (Figure 5).

### 3.6. EP Regulated the Myocardial Protein Expression of p-PI3K, p-Akt, Bad, Bcl-2, Bax, and Caspase-3

Western blotting analysis indicated that the EE group underwent a significant decrease in p-PI3K and p-Akt expression compared with the Con, EP, EP + EE, and LY groups. Compared with the Con group, the expression levels of p-PI3K and p-Akt increased dramatically. These results showed that EE inhibited the expression level of p-PI3K and p-Akt, whereas EP improved the expressions of p-PI3K and p-Akt. In the LY + EP + EE group, the expression of p-PI3K and p-Akt was obviously decreased compared with the EP + EE group, which showed that LY294002 inhibited EP-induced increasing expression of p-PI3K and p-Akt.

**Figure 6:**
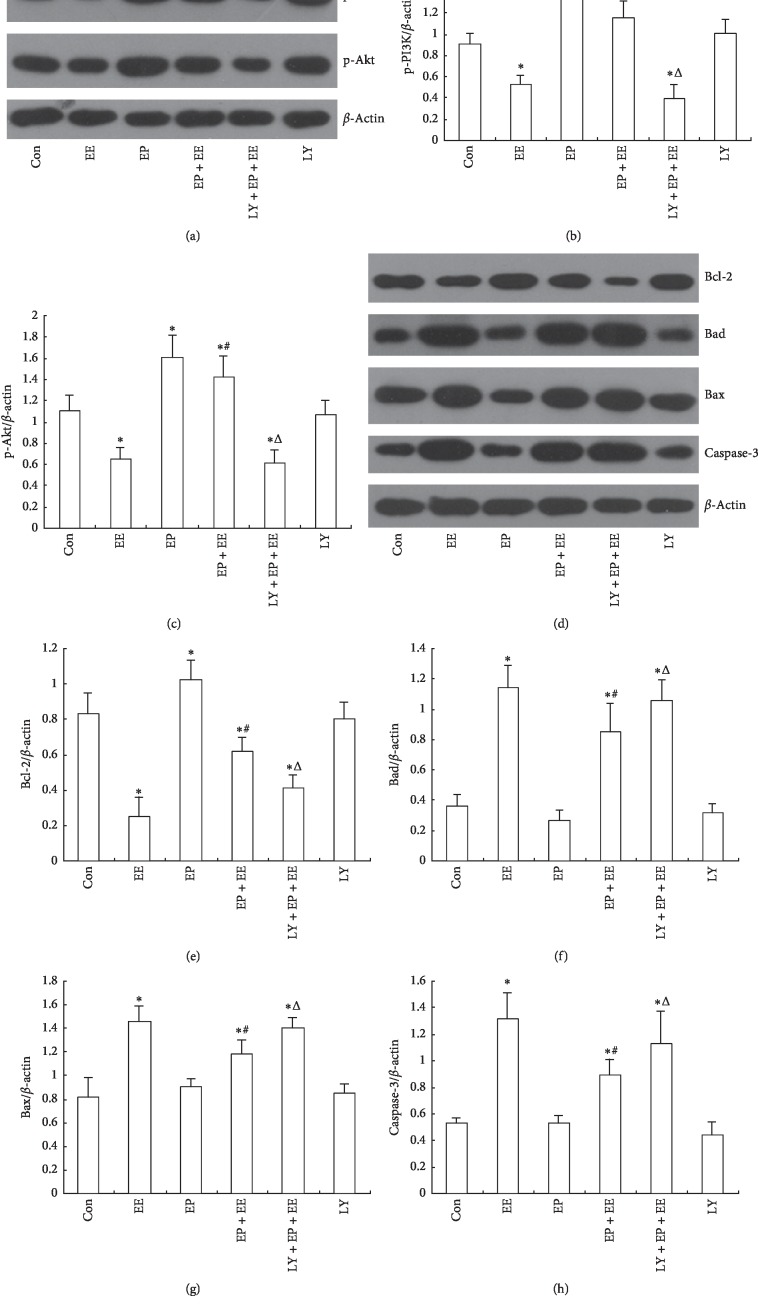
The effect of EP on the levels of p-PI3K, p-Akt, Bad, Bax, Bcl-2, and caspase-3 proteins in the rat myocardium after EE (*x* ± *s*, *n* = 6). For groups, see the footnote in Figure 1;(a) original recording showing the p-PI3K and p-Akt. (b) The ratio of p-PI3K/*β*-actin levels in the rat myocardium. (c) The ratio of nuclear p-Akt/*β*-actin levels in the rat myocardium. (d) Original recording showing the Bcl-2, Bad, Bax and caspase-3. (e) The ratio of Bcl-2/*β*-actin levels in the rat myocardium. (f) The ratio of Bad/*β*-actin levels in the rat myocardium. (g) The ratio of Bax/*β*-actin levels in the rat myocardium. (h) The ratio of caspase-3/*β*-actin levels in the rat myocardium. ^*∗*^*P* < 0.05 compared with the Con group. ^#^*P* < 0.05, EP + EE group compared with the EE group. ^Δ^*P* < 0.05, LY + EP + EE group compared with the EP + EE group.

Compared with the Con group, the EE group showed a significant increase in Bad, Bax, and caspase-3 expression and a significant decrease in Bcl-2 expression, whereas in the EP + EE group, the expressions of Bad, Bax, and caspase-3 proteins were significantly increased and Bcl-2 protein was significantly reduced. It showed that EE increased the proapoptotic protein expression and decreased the antiapoptotic protein expression in the myocardial tissue, while EP improved the antiapoptotic protein expression and reduced the proapoptotic protein expression, which indicated that EP inhibited cardiomyocytes apoptosis which was induced by EE. However, this antiapoptotic effect of EP could be weakened by the PI3K inhibitor; it was shown that the expression of Bad, Bax, and caspase-3 protein increased obviously, and Bcl-2 protein expression decreased obviously in the LY + EP + EE group (Figure 6).

### 3.7. Analysis of the Correlations between Cardiac Function Parameters and the Expression Levels of p-PI3K, p-Akt, Bad, Bcl-2, Bax, and Caspase-3

In the Con group, Pearson's correlation coefficients for mPTP and the level of the Bad protein with SW was −0.818 and −0.886 (*P* < 0.05). In the EE group, the mPTP open levels positively correlated with the Tau, and Pearson's correlation coefficient was 0.944 (*P* < 0.01). In the EP group, the level of the Bax protein positively correlated with the EDPVR and Pearson's correlation coefficient was 0.916 (*P* < 0.05). The level of the Bcl-2 protein was positively correlated with the d*p*/d*t*_max_ (*r* = 0.890, *P* < 0.05). Pearson's correlation coefficient between Caspase-3 and the CE was −0.885 (*P* < 0.05), which indicated a negative correlation. The level of the Bad protein positively correlated with the EDPVR (*r* = 0.996, *P* < 0.01). In the EP + EE group, the level of the Bax protein negatively correlated with the Ea and Pearson's correlation coefficient was −0.962 (*P* < 0.01). The p-Akt level was positively correlated with ESPVR (*r* = 0.943, *P* < 0.01). The p-PI3K level was positively correlated with CE and ESPVR (*r* = 0.996, *P* < 0.01 and *r* = 0.812, *P* < 0.05). In the LY + EP + EE group, the Bax level and the Caspase-3 level were negatively correlated with ESPVR (*r* = −0.955, *P* < 0.01 and *r* = −0.816, *P* < 0.05). The p-Akt level positively correlated with the dp/dtmax, with a correlation coefficient of *r* = 0.862 (*P* < 0.05).

## 4. Discussion

The present studies demonstrate that EP protects the heart against EE-induced injury as evidenced by reducing the injury of cardiac microstructure and ultrastructure, improving the cardiac function and cardiac efficiency and reducing myocardial apoptosis. We provided the first detailed key mechanisms that EP inhibited which include the mitochondrial pathway to reduce the myocardial apoptosis by activating the PI3K-Akt signaling pathway of cardiac damage by using the established rat model. The present studies indicated that for people who often engage in high-intensity exercise, EP allows the heart to elicit adaptive responses to EE. Moderate exercise preconditioning could reduce the heart damage which resulted from following intense exercise.

Exhaustive exercise could cause myocardial cell edema and increased membrane permeability, and the rupture of myocardial fibers caused the release of cTn-I in cardiomyocyte to peripheral blood [21]. The acute overload exercise increased the level of cardiac calcium protein in the peripheral blood, and the increase of the serum myocardial injury marker enzyme was related to exercise intensity [22]. After EP, the content of myocardial enzyme in serum of exhausted rats decreased, which indicated that EP had the ability to resist the myocardial injury. The present study describes left ventricular (LV) pressure and volume relations in detail and provides characterization of cardiac function. After EE, impaired inotropic state of the heart resulted in a suboptimal transfer of blood from the LV to the periphery with more excessively decline in SV, CO, and EF, which further influenced Ves and Ved, and lead to compensatory increase in HR. This study showed that EP could improve diastolic function and ventricular compliance in exhausted rats. The myocardium of the exhausted rats was damaged. The resistance of ventricular wall movement was increased, so PE increased and SW and CE decreased. Oláh et al. also demonstrated that CE and SW reduced in rats after exhaustive exercise [23]. However, the mechanical energy damage of the heart was alleviated after EP. Li et al. showed a significant impairment of cardiac function in experimental animals which subjected to exhaustive physical activity [24]. It has been reported that EE decreased the cardiac systolic function and impaired the cardiac mechanical energy, whereas it failed to observe any significant effect in cardiac diastolic function [23]. These conflicting data may result from different experimental protocols, such as the speed, exercise training mode, interval time, and differences in exercise protocols. In this study, the alterations of hemodynamic parameters indicated that EP played a role in protecting against EE-induced deteriorated inotropic state of the LV. EP alleviated the EE-induced myocardial dysfunction. EP exerted cardioprotective effects for EE rats.

The PI3K-Akt pathway played an important role in regulating cell apoptosis. The mitochondria-dependent apoptotic pathway was mediated by Bcl-2, Bad, and Bax [12]. Bcl-2 prevented apoptosis by inhibiting the activation of the proapoptotic family member Bax [25]. Bax affected the permeability of the mitochondrial membrane by acting on the mPTP [26]. Phosphorylation of Bad alleviated the inhibitory effects of Bad on prosurvival protein Bcl-2 [27]. Some previous studies have shown that exercise training increased Bcl-2 in myocardial tissue [28, 29]. In the current study, we found EP-induced activation of the PI3K-Akt pathway contributed to increase the level of Bcl-2 and decrease the levels of Bad, Bax, mPTP openness, and caspase-3, so EP resisted the cardiomyocytes apoptosis caused by EE. From the results of the correlations, we could learn that, in the EE and EP groups, the increased mPTP open levels and Bax levels impaired the diastolic function of the heart. The PI3K-Akt pathway exerted a protective effect on the heart and improved the cardiac function parameters. The specific PI3K-Akt signaling pathway blocker LY294002 was applied to test this protective effect. EP-induced beneficial effects on exhausted hearts was attenuated. Therefore, the cardioprotective effect of EP might inhibit apoptosis by activating the PI3K-Akt signaling pathway. It is suggested that the activation of PI3K-Akt channel might play an important role in the inhibiting the apoptosis mechanism of EP.

PI3K-Akt is closely related to cell life activities. It is involved in the regulation of important physiological functions such as cell activation, protein synthesis, and mediates numerous external stimulus signals to induce cell growth, differentiation, survival, apoptosis, and malignancy. Akt activates directly on multiple targets such as the apoptotic family of Bcl, nuclear transcription factor-*κ*B (NF-*κ*B) and nitric oxide synthase (NOS) and inhibits apoptosis by reducing open mPTP [30]. But, the mechanism of myocardial apoptosis is complex and involves multiple targets; whether EP reduces the myocardial apoptosis in exhausted rats through NF-*κ*B and NOS remains to be further explored. In addition, the PI3K-Akt pathway is also associated with autophagy and inflammation, which suggests that EP may play a protective role in exhausted rats through these mechanisms, but we need to explore it further [31, 32].

In summary, EP reduced the myocardial injury in the exhausted rats. It not only reduced the release of CK-MB and cTn-I in serum, but also increased myocardial systolic and diastolic function and improved the cardiac efficiency. By activating the PI3K-Akt signaling pathway, EP inhibited the mitochondrial pathway to reduce the myocardial apoptosis.

## Figures and Tables

**Table 1:** The effect of EP on the cardiac function parameters of exhausted rats.

	Con	EE	EP	EP + EE	LY + EP + EE	LY
Parameter						
CO (ml/min)	50.78 ± 4.35	42.04 ± 3.70^*∗*^	54.95 ± 4.05^*∗*^	46.28 ± 3.89^*∗*#^	42.15 ± 3.62^*∗*Δ^	51.03 ± 3.20
SV (*μ*L)	136.25 ± 17.22	105.44 ± 10.77^*∗*^	150.38 ± 12.03^*∗*^	121.38 ± 14.03^*∗*#^	107.25 ± 10.77^*∗*Δ^	131.75 ± 16.51
HR (bpm)	374.50 ± 31.78	401.75 ± 22.75^*∗*^	349.63 ± 17.52	388.38 ± 22.04	395.50 ± 23.84	367.63 ± 29.48
Ves (*μ*L)	101.25 ± 9.82	140.13 ± 13.27^*∗*^	86.25 ± 12.31^*∗*^	126.54 ± 10.79^*∗*#^	142.04 ± 17.82^*∗*Δ^	97.88 ± 13.00
Ved (*μ*L)	212.50 ± 11.14	236.50 ± 20.95^*∗*^	196.25 ± 12.88^*∗*^	220.00 ± 16.74^#^	229.88 ± 12.22^*∗*^	215.50 ± 15.46
Pes (mmHg)	121.50 ± 15.34	97.38 ± 10.65^*∗*^	123.50 ± 17.25	111.00 ± 12.28^#^	103.00 ± 7.96^*∗*^	118.38 ± 13.76
Ped (mmHg)	3.83 ± 0.58	4.56 ± 0.33^*∗*^	3.42 ± 0.53	4.22 ± 0.37	4.49 ± 0.43^*∗*^	3.66 ± 0.45
Systolic indices						
EF (%)	62.98 ± 7.44	43.50 ± 5.80^*∗*^	71.86 ± 11.92^*∗*^	53.25 ± 9.45^*∗*#^	44.38 ± 3.76^*∗*Δ^	60.86 ± 10.91
d*p*/d*t* max (mmHg/s)	8922.38 ± 983.83	7211.50 ± 658.11^*∗*^	9733.75 ± 780.49^*∗*^	8091.50 ± 691.04^*∗*#^	6929.38 ± 861.76^*∗*Δ^	8672.38 ± 774.45
ESPVR	2.50 ± 0.36	1.45 ± 0.12^*∗*^	2.85 ± 0.50^*∗*^	1.90 ± 0.19^*∗*#^	1.58 ± 0.20^*∗*Δ^	2.53 ± 0.30
Diastolic indices						
EDPVR	0.022 ± 0.004	0.037 ± 0.005^*∗*^	0.016 ± 0.005^*∗*^	0.031 ± 0.006^*∗*#^	0.035 ± 0.004^*∗*^	0.021 ± 0.003
Tau	9.57 ± 1.03	13.63 ± 1.95^*∗*^	10.03 ± 0.94	11.74 ± 1.83^*∗*#^	13.28 ± 1.76^*∗*Δ^	9.50 ± 0.85
−d*p*/d*t* min (mmHg/s)	6595.88 ± 544.95	5354.63 ± 527.54^*∗*^	7148.50 ± 608.64^*∗*^	6117.13 ± 509.85^#^	5517.13 ± 523.94^*∗*Δ^	6384.63 ± 555.96
Mechanoenergetics indices						
PE (mmHg ∗ *μ*L)	9916 ± 1665	13141 ± 2293^*∗*^	7679 ± 1210^*∗*^	10997 ± 1887^#^	12034 ± 2447^*∗*^	9779 ± 1691
SW (mmHg ∗ *μ*L)	16536 ± 2286	10875 ± 2506^*∗*^	20753 ± 2653^*∗*^	1396 ± 2273^*∗*#^	11591 ± 2010^*∗*Δ^	16285 ± 1824
CE (%)	61.88 ± 7.57	45.63 ± 5.95^*∗*^	72.38 ± 13.18^*∗*^	56.25 ± 7.13^#^	48.00 ± 5.07^*∗*Δ^	63.12 ± 7.35

## Data Availability

All datasets analyzed to support the findings of the current study are available from the corresponding author upon reasonable request.
